# “I Agree to Disagree”: Comparative Ethical and Legal Analysis of Big Data and Genomics for Privacy, Consent, and Ownership

**DOI:** 10.7759/cureus.18736

**Published:** 2021-10-13

**Authors:** Seema Belani, Georgina C Tiarks, Neil Mookerjee, Vijay Rajput

**Affiliations:** 1 College of Allopathic Medicine, Nova Southeastern University Dr. Kiran C. Patel College of Allopathic Medicine, Fort Lauderdale, USA; 2 College of Allopathic Medicine, Nova Southeastern University Dr. Kiran C. Patel College of Allopathic Medicine, Davie, USA; 3 Medical Education, Cooper Medical School of Rowan University, Camden, USA; 4 Medical Education, Nova Southeastern University Dr. Kiran C. Patel College of Allopathic Medicine, Fort Lauderdale, USA

**Keywords:** security, privacy, ownership, consent, legal, ethics, genomics, big data, bioethics

## Abstract

Statement of Purpose: Digital healthcare, as it relates to big data and genomics, presents a real threat to privacy and ownership rights for individuals and society.

Research Question/Hypothesis: Our experience with genomics provides a lens to facilitate the way we navigate toward a future health data space. Contemporary and innovative legal and ethical models can be applied to concepts of privacy, ownership, and consent in relation to big data.

Significance: Technological innovation has transformed healthcare at a faster rate than legal reform, security measures, and consent policies can adapt. The Health Information Portability and Accountability Act (HIPAA) has been recognized as a work in progress, with respect to big data as it relates to healthcare and individual wellbeing. The shortcomings of HIPAA, and its application to big data, can be paralleled with its prior limitations surrounding genomics in the last two decades. The Genetic Information and Nondiscrimination Act (2008) and Genomic Data Sharing Policy (2015) were established to overcome HIPAA’s inadequacies concerning genetic discrimination and security. These policies can serve as a basic model for our approach to legislative reform as it relates to privacy risks with big data generated in healthcare and from healthy individuals in society who are not patients. In addition to notions of privacy, concepts of ownership and consent have become increasingly vague and opaque. The technological advancements have facilitated access and transmission of information, such that big data can be sold for financial gain for commercial enterprise. This applies to genomics, with companies like 23andMe, in addition to big data, as it relates to big tech giants like Apple or Google who oversee wearable and search term data. Clarity of ownership within a digital healthcare arena needs to be defined through ethical and legal frameworks at a global level.

Approach: A narrative review of the literature published between 2010 and 2021 was performed using PubMed and Google Scholar. Articles discussing privacy, security, ownership, big data, and genomics were included as relevant literature.

Importance: As a society, we are at a crossroads; we must determine the extent of privacy that we are willing to give for science and society. We cannot continue with the current status quo in hope that individual will be used for the greater good of society. We need to strive for a cohesive approach to combat privacy violations by encouraging legislative reform, ethical accountability, and individual responsibility.

## Introduction and background

Introduction

The definition of privacy has evolved from its traditional image of secrecy or concealment [1]. Over the years, both the Health Insurance Portability and Accountability Act (HIPAA) Privacy Rule and the process of informed consent have evolved to protect both autonomy and privacy in healthcare. However, the current HIPAA and consent process have not been able to adjust with rapid advancements in big data and genomics in the last two decades. The growth of the internet and technology facilitate information sharing, which has driven a shift toward a new model of privacy. This model allows for individual control over the extent to which information about themselves is communicated to others [1-2]. Nevertheless, privacy breaches can occur when access to information occurs by the wrong party, through an unauthorized process, or for inappropriate reasons [3]. As we move toward a digital healthcare that leverages genomics and big data, privacy risks are a real threat to both individuals and society at large. And although US privacy laws have been developed for both health data and genomics, they approach information in a way that is different than clinical data and is dependent on who creates and handles the data [3]. Can our previous experience with genomics guide the way we think about the future health data space? Legal and ethical frameworks can be applied to draw similarities and highlight differences between the two areas concerning concepts of privacy, ownership, and consent.

Background

Genomics is the study of an individual’s entire genome or gene sequences. The field strives to clarify how genes interact with other genes, the environment, and psychosocial factors. Genomic data is acquired through clinical samples, such as blood, saliva, or tissue biopsies. Genomic research has evolved and commercialized in recent years. Initially, specimen samples were primarily collected only for clinical research projects or making genetic diagnoses and treatments. However, now law enforcement and direct-to-consumer (DTC) genetic testing use genomics data in the private market for prediction of clinical conditions and ancestry.

Big data, conversely, is defined by “three Vs”: volume, velocity, and variety, and in the context of healthcare should be visualized as an ecosystem. It includes not only “small data” housed within traditional patient health records, but also larger health data generated by people or products outside of the traditional healthcare system [3]. For example, vital signs collected by wearable devices, symptom-related Google searches, or Amazon shopping behaviors constitute user-generated information that permits inferences about health and related behaviors.

Health privacy and its associated challenges should elicit a distinct and important comparison between the privacy framework used by both genomics and big data. At face value, both genomics and big data deal with health information that concerns the individual, and to some extent, the larger community. Yet, these advancements have not only created significant privacy risks due to exposure, but also allowed for unprecedented concerns for human dignity: the innate right for individuals to be treated with respect and liberty as given in the US constitution.

## Review

Current legal framework for privacy

Breakthroughs in technology have brought wearable devices and medical smartphone applications into the healthcare arena. As technology continues to advance, laws and regulations have significantly fallen behind to protect patients and consumers. The Health Insurance Portability and Accountability Act (HIPAA) Privacy Rule was passed in 1996 to protect individuals’ health data [4]. At that time, there were only around 20 million individuals who had access to the internet worldwide [5]. There were only approximately 100,000 websites, the Human Genome was still under development, and Google was non-existent [5]. HIPAA has received several modifications since it was originally developed. This includes the addition of electronic medical records (EMR) with the most recent update in 2013 [4]. However, although HIPAA is the primary health-related privacy law, it has yet to undergo amendments that will cover big health data from recent technological advances. A 2017 review indicated that there were approximately 300,000 health apps available, demonstrating the vast amount of data that is being collected. Additionally, in 2018, 17 billion smartphone and tablet users used mobile health apps [6-7]. This is a far cry from the world HIPAA was built for in 1996. In fact, prior research has noted that HIPAA was not created as a “one and done law” and should be seen as a work in progress [8]. While mobile health apps and wearable devices offer considerable benefit by providing on-the-go electrocardiograms, heart rate monitoring, calorie counting, etc., legal protection related to privacy and ownership of this large-collected data may be lacking. HIPAA’s regulations to protect health data applies to all “covered entities” [9]. The term “covered entities” is meant to refer to anyone operating within healthcare, which includes healthcare providers, hospitals, and insurance companies. Yet now, big technology companies like Google, Apple, and Fitbit dominate a new health data sector composed of private wearable devices and smartphone apps, which are not covered under the original privacy laws [3]. In addition, HIPAA does not protect user-generated data created from online medical searches or health apps that track data like blood sugar [3]. Overall, the data that is generated outside of the standard medical records or healthcare system is not subject to the same privacy laws under HIPAA [3]. Though there are other regulations in place to protect data like the Computer Fraud and Abuse Act and the Electronic Communications Privacy Act, these rules may not encompass private health apps [7].

While wearable devices and technology are advancing rapidly, regulations and laws are not moving at a similar pace [2]. The current inadequacies of HIPAA in big data are parallel to the shortcomings decades ago regarding regulations surrounding genomics, genetic testing, and treatments. Although HIPAA provided some protections, there were several limitations [10]. Among other things, it did not ban genetic discrimination of individual plans or prevent insurers from necessitating genetic testing to receive coverage [10]. To overcome these loopholes, the Genetic Information and Nondiscrimination Act (GINA) was passed in 2008, which protected against health insurance discrimination [10]. Until GINA was established, fundamental genetic protections were missing [10].

De-identification and re-identification in the context of privacy and big data

Further shortcomings of legal regulations in healthcare are anonymization and confidentiality. In theory, anonymization occurs when no one ever knows the individual’s identity. In the context of healthcare, anonymity was traditionally considered an irreversible action before discovering the reality and facility of re-identification measures. Confidentiality, on the other hand, is described as information that is only available to authorized individuals [2]. Given these definitions, confidentiality can be ensured when privacy is attained. This is problematic because the public is supportive of health information sharing for research purposes, under conditions of privacy assurance and de-identification; however, de-identification is not a sure means to achieving privacy [11].

With regards to medical data, HIPAA has loopholes; it protects patients by de-identifying data through the removal of 18 identifiers, but the data can often be re-identified using data analysis by combining multiple data sets [3]. To put this in perspective, voter rolls contain the name, address, ZIP code, birth date, and sex of every voter within a given city. When this information is analyzed with reference to health insurance data and with health record data that has been scrubbed of protected health information, it is very possible to determine an individual’s prescriptions and medical history [3]. Big data expands the pool of information, further increasing the vulnerability to re-identification. When these data sets are combined with internet search queries, social network data, or even facial recognition, anonymity can be easily lost.

In genomic research, information is de-identified to protect both the individual and their family members. In the early stages of genomics, there was a fear of re-identification due to its unique nature. However, reidentification at that time was considered unlikely because the only source of identified genetic samples were forensic databases. These databases were of little value because they relied on short tandem repeats, and consisted of a limited sample, only accessible by law enforcement [1]. Over time sequencing and storage costs have decreased, which has led to the emergence of companies like 23andMe or Ancestry.com that offer direct-to-consumer DNA testing. These companies analyze thousands of single nucleotide polymorphisms (SNPs), and as a result, their samples are incredible in size. Furthermore, many of these companies house their genetic databases on open-access websites. These factors allow for genetic data to be readily re-identified. In response to the reidentification threats posed by open-access sites, The National Institutes of Health (NIH) shifted toward controlled-access gene banks in 2015. The NIH established the Genomic Data Sharing Policy (GDS), which requires all NIH-funded research to submit de-identified human genomic and phenotypic data to databases selected by the NIH [12]. While there are limitations within this policy, i.e., the policy applies only to NIH-funded research, it nonetheless serves as a model for security measures.

Blockchain technology is a prime example of a means to overcoming some of the privacy and security challenges. Research concerning the application of this technology in the healthcare arena predicts features of security, authentication, and decentralized storage will be of most value [13]. In terms of security, blockchain utilizes a decentralized ledger, which allows for information storage on multiple sources versus one [13]. Furthermore, with respect to authentication, it applies a model of a private key, which is tied to a public key. Users who wish to create, alter, or even view data stored within the blockchain require a private key [13]. However, while blockchain has established utility in banking, finance, real estate, and government, it is just now gaining momentum in the healthcare sector [13]. With more attention and focus, blockchain can be used to solve some aspects of security for both big data and genomics.

Ethics of ownership and consent

Ownership can be described as, “… both the possession and responsibility for information” [14]. However, over the past several years “ownership” as a term is becoming increasingly vague. Recent advancements in technology have led to easier access and transmission of information. Due to the simplicity of data transfer, more data may be sold by various organizations for financial gain. They might be sold to commercial companies such as Amazon, which can create questions when examining who owns the data [15]. Once this data has been sold, the patient’s data is then owned by the company that bought the data. This can all occur without communicating at all to the patient. Amazon is also not the only company to spotlight; other big tech giants like Google or Apple can potentially utilize search engine behavior, ads, or mobile health data in ways that result in an opaque understanding of who truly owns the data.

Another big issue that arises when dealing with ownership is the process of consent in giving information to a particular company or third party. When a user engages with a program for the first time, the first pop-up is almost always a consent form. However, in many instances these users scroll and press “I agree” without reading the whole consent form. Every time “I agree” is pressed, ownership is declined from that user. If the users’ data becomes part of a data set, ownership declines even more [3]. The broad terms outlined in consent forms also contribute to a hazy definition of ownership. In one study, more than half of the people who participated were not familiar with the application’s security policies [2]. This indicates how easily ownership may be transferred without users knowing what they are giving up about their own data. One example of this applies to wearable devices. People accept consent agreements thinking that the company is only storing health information gathered on the wearable. However, these companies are receiving much more than that. For example, a company’s program called Strava has a heat map feature where the location of individuals using the fitness tracking application can be revealed. To put this in a larger context, US soldiers who used Strava had their movements revealed while in Syria. It also revealed the troops’ movement in Syria. This is just one example of how pressing “I agree” to a dense consent form containing broad terms can obscure ownership. The problem with consent will become part of the future, unless there is significant policy and/or legal changes. One reform approach is for companies to consider layman language when writing their consent forms. Currently, consent forms are lengthy and contain dense, broad terms, which isn’t user-friendly. In fact, this design not only deters the user from actually reading the consent form, but also draws the user to quickly scroll to the bottom to simply press “I agree.” One study illustrated that more than half of the subjects were neither clear about how their wearable device security information policies worked nor how they protected their information [2]. In addition, more than half of the study’s participants were not clear about how the wearable devices information was transmitted, stored, and labeled [2]. A simple, easy-to-read consent form would have solved these issues.

Ownership has also lacked clarity in the realm of genomics. DNA testing has skyrocketed since the human genome project was completed. Numerous companies such as 23andMe provide a service for individuals to have their genome sequenced. This new phenomenon brings up issues about ownership for both the individual getting tested and their relatives, since they share similar genetics. One study demonstrated how easily this can be done by cross-matching information from the internet with basic demographic information that is not covered by HIPAA. The study also found that surnames can be derived from short tandem repeats of the Y chromosome (Y-STRs). This information is freely accessible through public search engines such as PeopleFinders.com. This study illustrated how easily relatives and their information can be identified by using one person’s surname along with the year of birth and residency [16]. Although that family member did not give consent, their data was made available because a genetic relative received DNA testing. This is just one example of how ownership loopholes exist, which are becoming more apparent with the advancement of science and technology. As more people begin participating in DNA testing services, this problem will only continue to grow.

Discussion

We are at a pivotal moment in society; individuals must determine the extent of privacy they are willing to give. Should society continue with the current status quo and agree to long consent forms, open-access gene banks, and weak security measures with hope that individual health data will be used for the greater good? Or should society lean on the government to enact laws and regulations that hold companies socially accountable? The next direction and steps are critical toward moving the needle toward better privacy, similar to the information on genomics.

Legislative Level

Legal reforms on statewide, national, and global levels are a potential solution to account for the modern-day privacy barriers that were not built into current regulations. With fewer than half of World Health Organization (WHO) countries institutionalizing private health data privacy regulations, there is an obvious need for a worldwide conversation [17]. While the United States relies largely upon HIPAA to protect its health-related data, other countries have sought to enact new regulations to cover the advances in big data. The European General Data Protection Regulation (GDPR), which was enacted in 2016, is often referred to as a gold standard that can act as a guideline for future amendments [18]. The GDPR outlines that any company or organization is subject to scrutiny as a “controller” or “processor” [18]. Unlike HIPAA, these companies do not need any healthcare affiliation to fall under this privacy law. Additionally, protected personal data is determined to be any information related to the individual and not simply health-related data [18]. California has also enacted a new regulation as of 2020 that uses the GDPR as a template. Yet, their regulation, the California Consumer Privacy Act (CCPA), goes further to specify that the collection and sale of personal information is also protected and “personal information” accounts for any information linked to a person or their household [18-19]. These new laws are just a few examples of the amendments that can be introduced. Incentives can also be given to companies for responsibly handling data. Moreover, governments may want to promote personal responsibility and consumer education so that individuals understand what they are consenting.

Company Level

While legal reform can theoretically be leveraged to incentivize companies for responsible data handling, it is ultimately the company’s decision to ensure security. Legislation aside, it is typically in the company’s best interest to develop strong security measures because healthcare data is worth more on the black market than credit card numbers. Companies that then house healthcare data become attractive to hackers, and that whole company as a result becomes vulnerable. Thinking beyond a single health record data breach, a company that becomes a victim of a cyber-attack can expect significant repercussions such as negative press coverage and undesirable lawsuits. Negative press coverage can also have a profound effect on a company’s long-term reputation. This kind of feared damage should motivate companies to build proper security measures before confronting the anticipated downstream consequences.

Individual Level

Although legal reforms are great in the long term, it is unrealistic to assume legislation and company accountability will happen overnight. In the meantime, each individual has to determine the extent of privacy that they are willing to share. One option is to continue with our current status quo of long consent forms, weak security measures, and minimal legislation in the hopes that this information will be used for the greater good of society. Having access to a plethora of data may help further research to find new treatments, cures, and improve individualized medicine [20]. On the other hand, these privacy violations may have a negative impact on the way society views future advancements. Encouraging individual responsibility may be a swift solution to privacy concerns. Many individuals are unaware of the extent to which their privacy may be breached while using new technology and accepting consent forms. This may be combatted through educational initiatives aimed at persuading society to read consent forms. Social media is a relevant example of a medium that may play a role in creating a youth movement, which motivates individuals to learn more about what privacy they are giving up by accepting consent forms and using certain technologies. We should push back at companies who attempt to trick consumers into signing away their rights through extensive and intricate language. Likewise, society may take another step forward by boycotting companies that have minimal security measures, vast consent forms, and access more data than is necessary.

## Conclusions

Parallels can be drawn between the lifecycle of genomics and big data in the context of healthcare privacy and security. The genomic legislative reform that we have seen through GINA and GDS serve as a platform for how the field can approach privacy risks in the context of big data in healthcare. Until then, it is up to individuals to speak up against privacy violations. Society at large needs to shift focus to combat privacy and security issues by encouraging swift legal reform, company accountability, and individual responsibility.
